# Supplementary material for: Design and characterization of hyperactive mutants of the Agrobacterium tumefaciens telomere resolvase, TelA
Source: PLoS One. 2024 Jul 25;19(7):e0307590. doi: 10.1371/journal.pone.0307590 (PMC11271964; doi:10.1371/journal.pone.0307590)
Supplement: S1 Raw images — (PDF) [file pone.0307590.s001.pdf]

S1 raw images

Fig 6 raw images

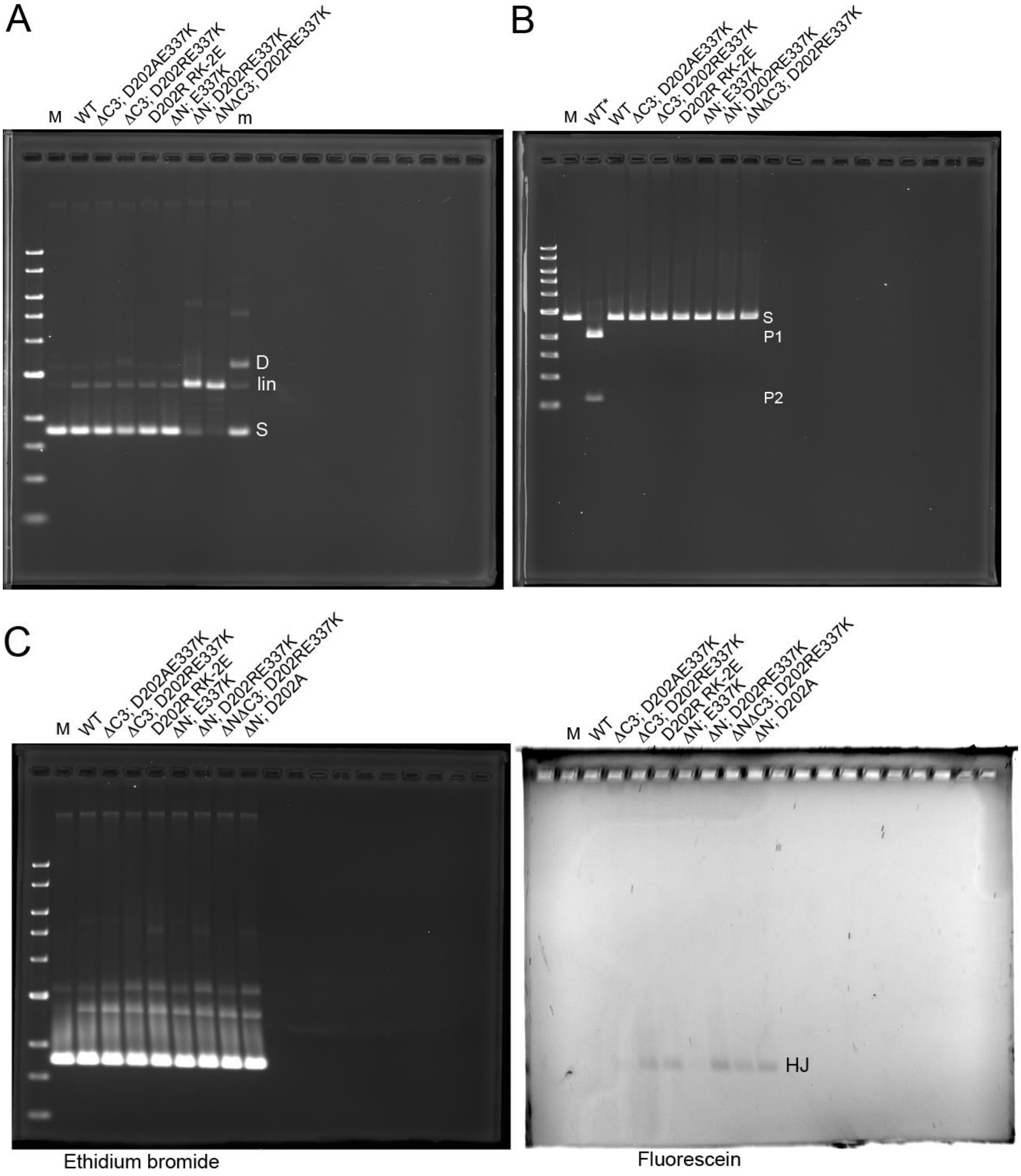

Uncropped gels for Fig 6.

Fig 7 raw images

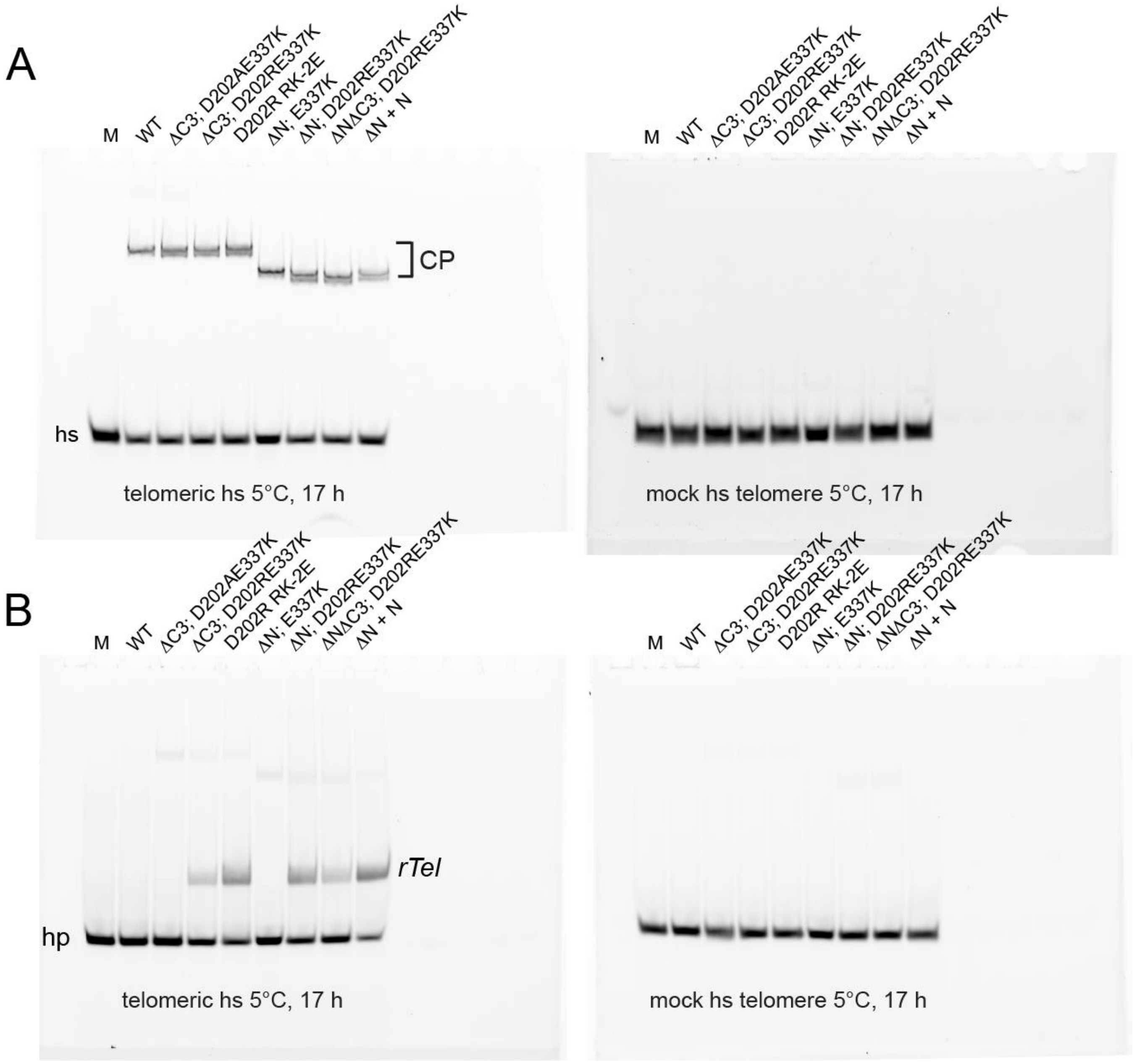

Uncropped gels for Fig 7.

S2 Fig raw images

B

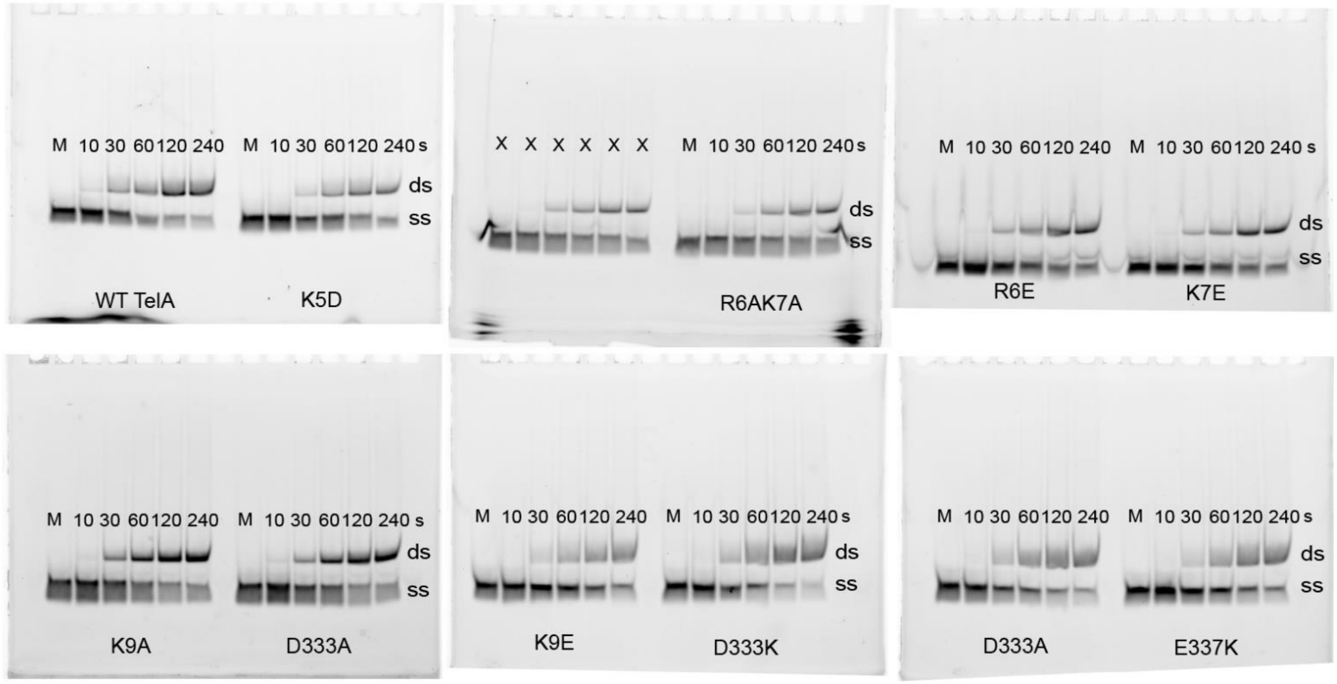

Uncropped gels for S2 Fig. Note that the gel panels are not presented in exactly the same order as in the original file. Where they appear X's mark lanes of mutants not reported in this study.

S3 Fig raw images

annealing; interface 2 mutants

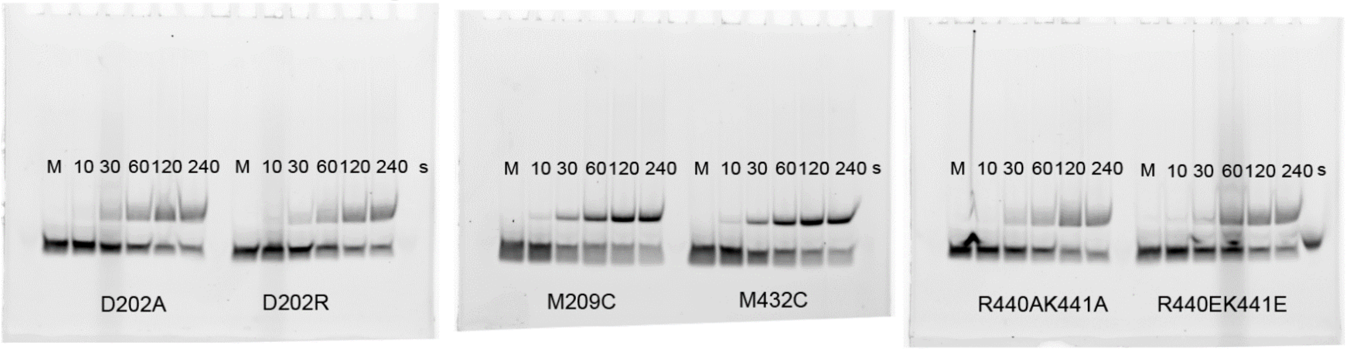

annealing; interface 1 + 2 mutants

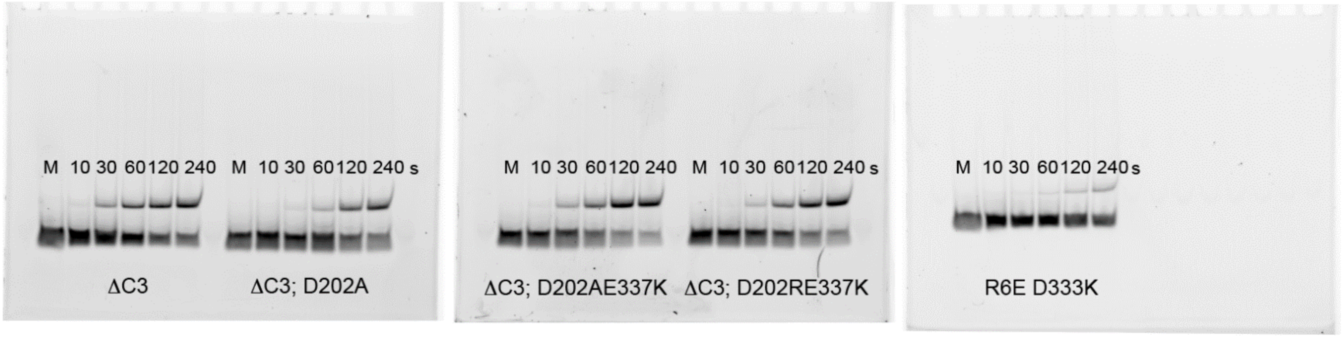

annealing; paired charge reversal interface 1

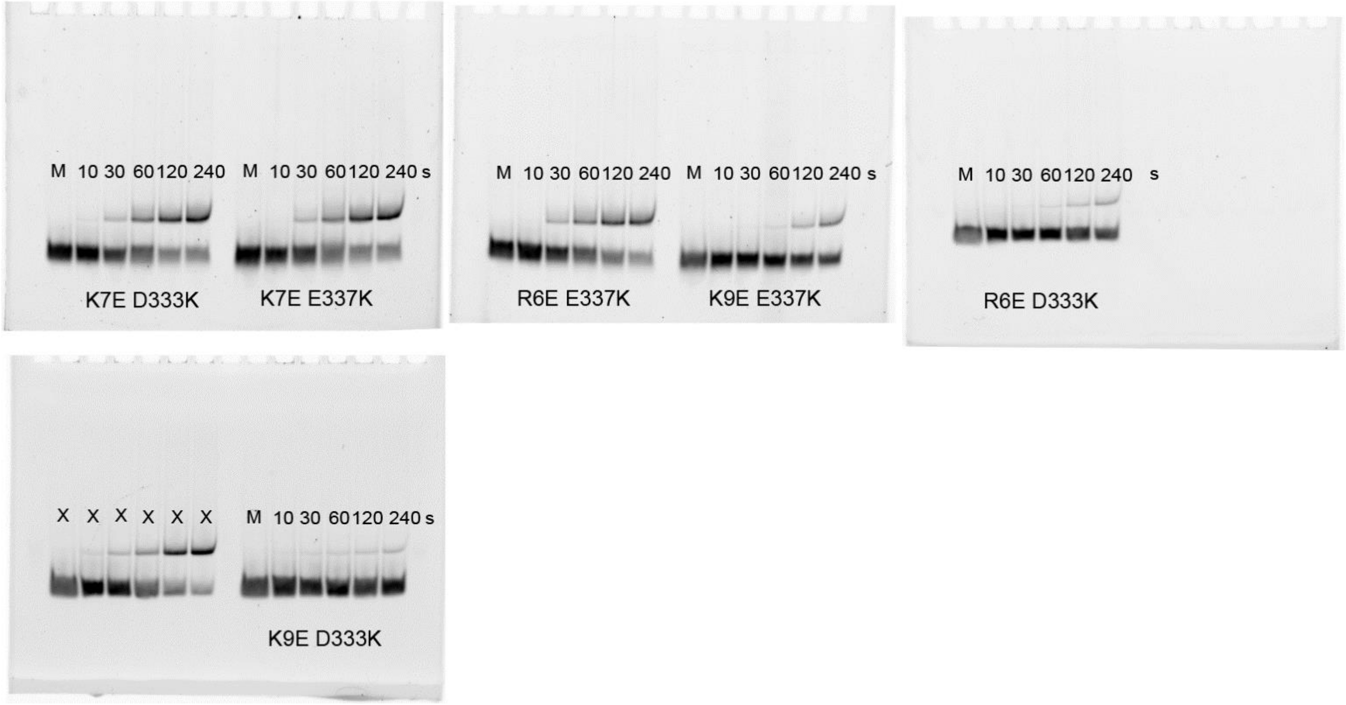

Uncropped gels for S3 Fig. Note that the gel panels are not presented in exactly the same order as in the original file. Where they appear X's mark lanes of mutants not reported in this study.

S4 Fig raw images

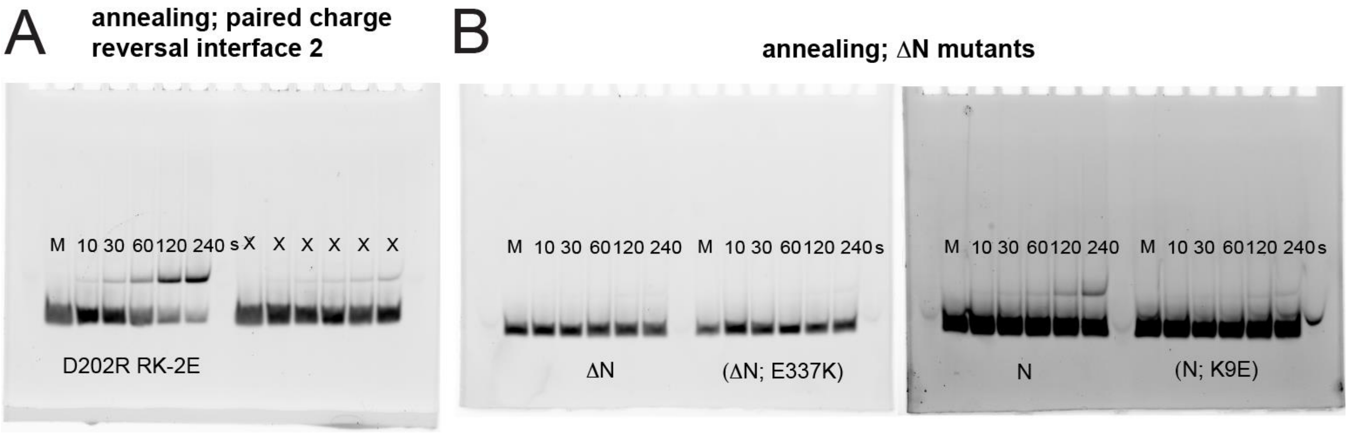

Uncropped gels for S4 Fig. Where they appear X's mark lanes of mutants not reported in this study.

S5 Fig raw images

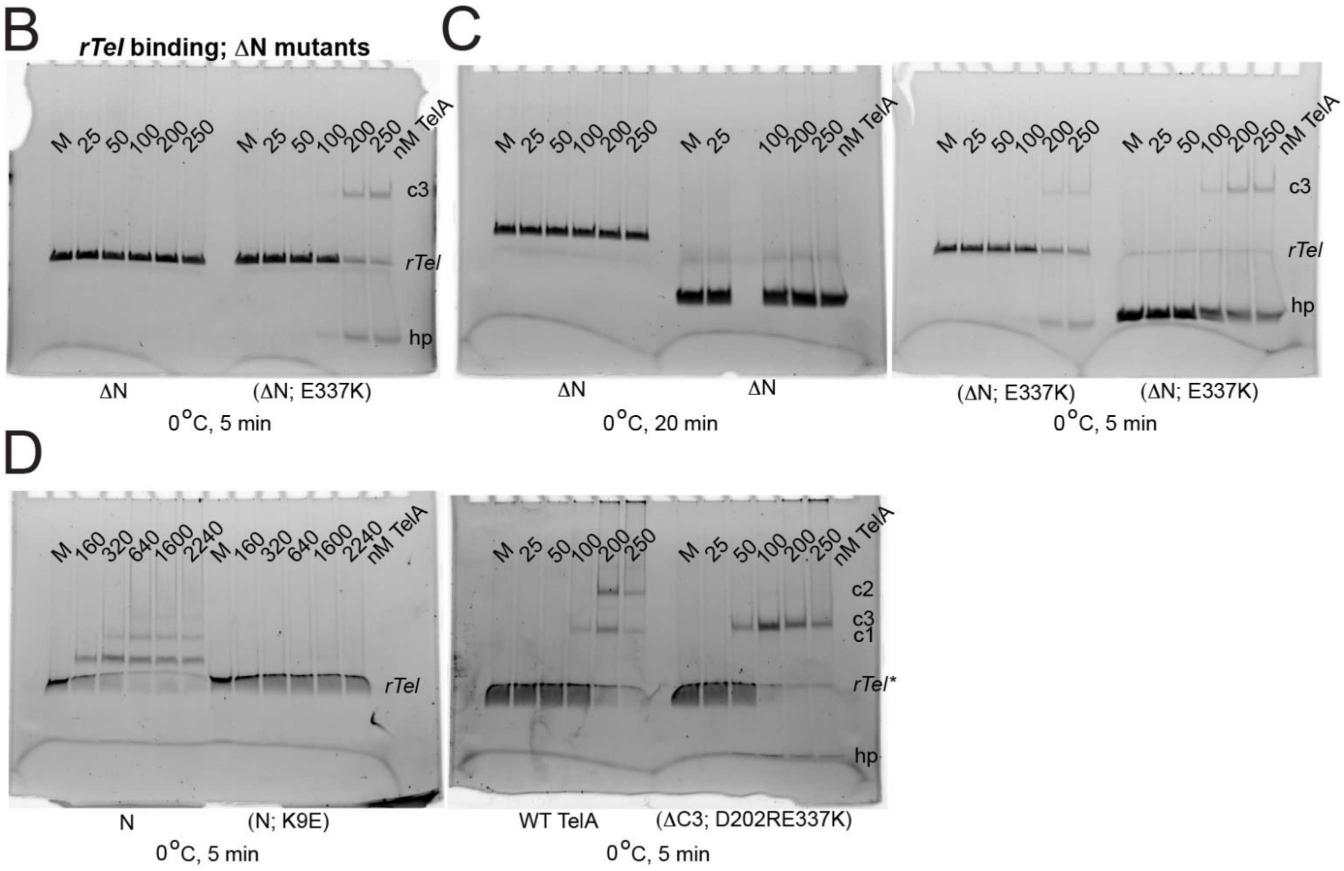

Uncropped gels for S5 Fig.

S6 Fig raw images

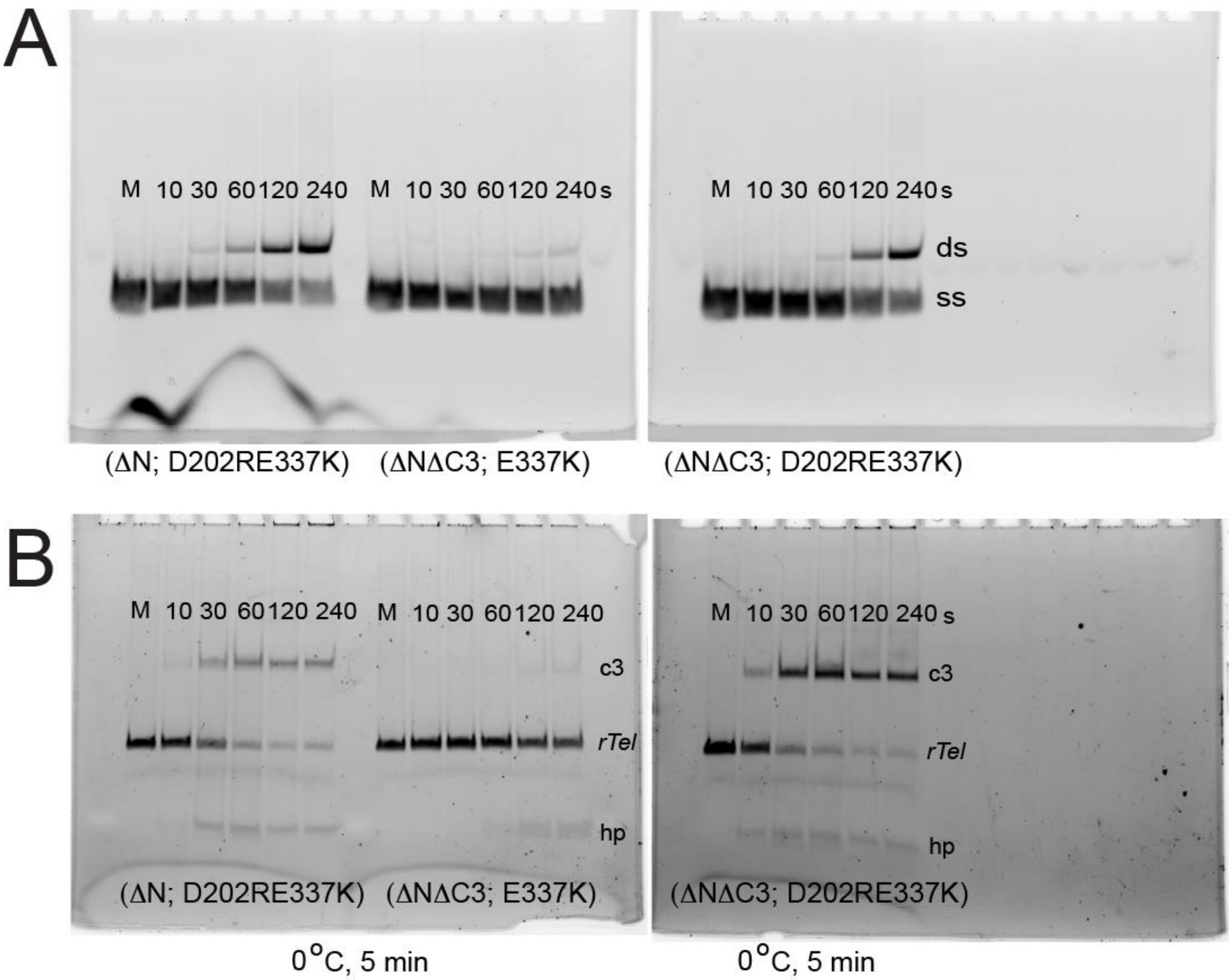

Uncropped gels for S6 Fig.

S7 Fig raw images

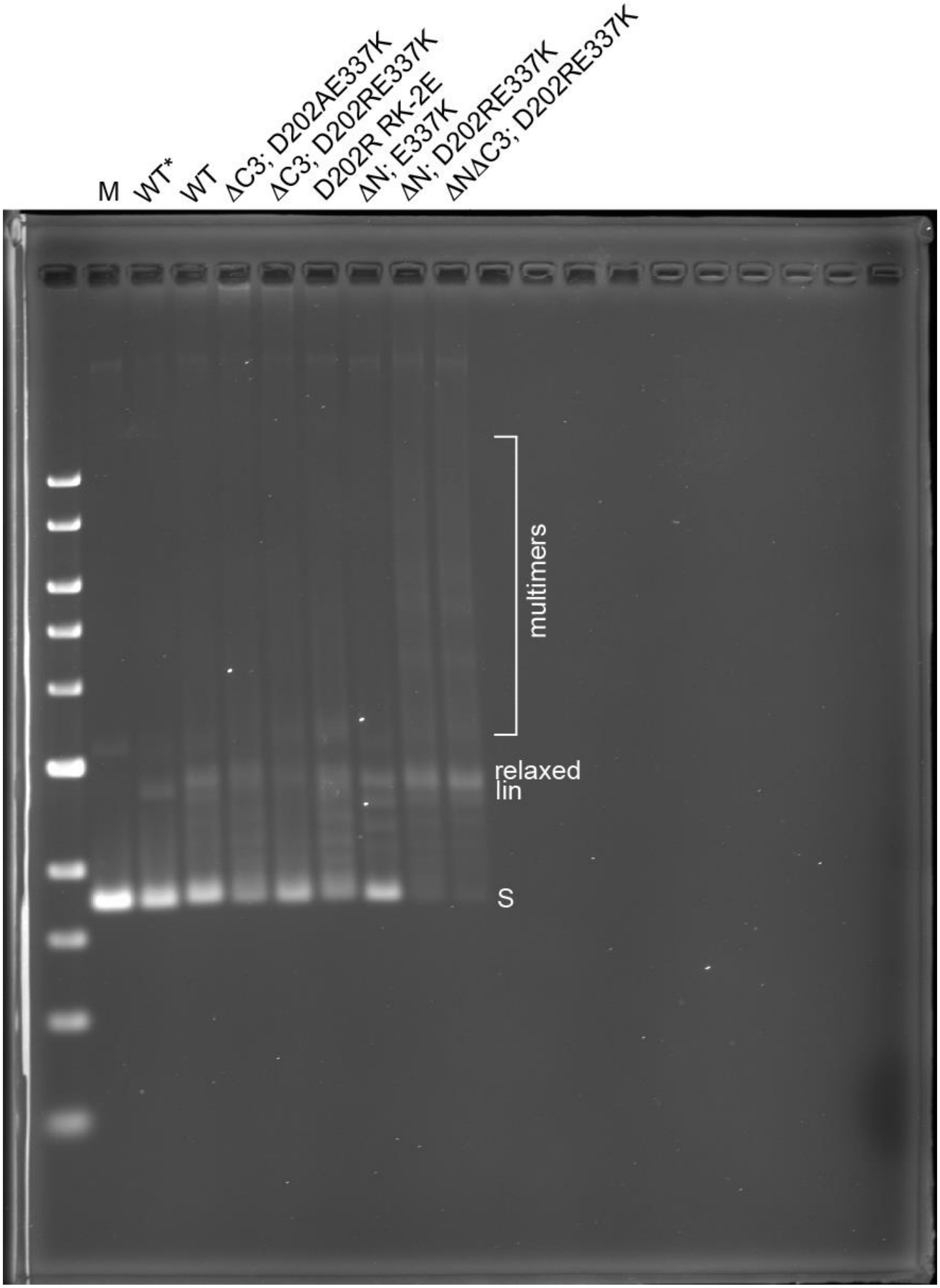

Uncropped gel for S7 Fig.
