# Supplementary material for: Design and characterization of hyperactive mutants of the Agrobacterium tumefaciens telomere resolvase, TelA
Source: PLoS One. 2024 Jul 25;19(7):e0307590. doi: 10.1371/journal.pone.0307590 (PMC11271964; doi:10.1371/journal.pone.0307590)
Supplement: S1 File — (PDF) [file pone.0307590.s002.pdf]

Supporting information

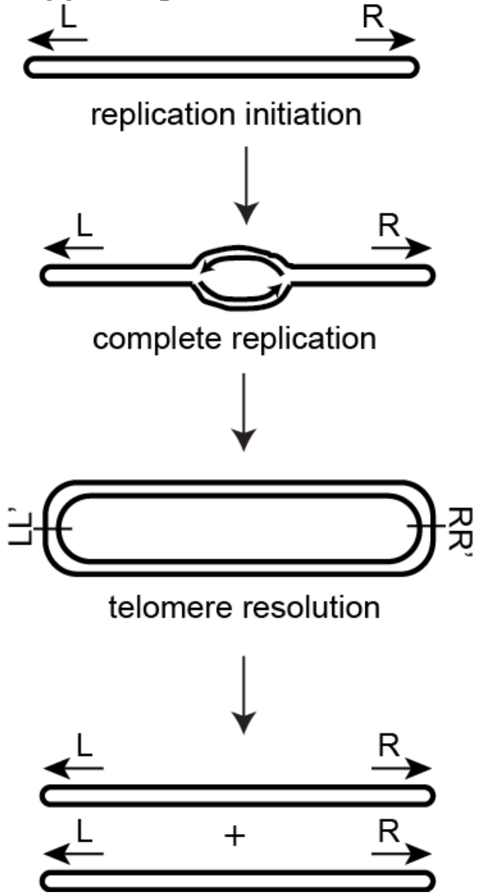

S1 Fig. Replication pathway for linear DNAs with hairpin telomeres.
Replication initiates internally at an *ori* and proceeds bidirectionally out towards the hp telomeres. Somehow, the replication machinery completes replication through the hp telomeres producing an inverted repeat circular dimer intermediate. A DNA breakage and reunion reaction at the replicated telomere junctions (*rTel*s; LL' and RR') called telomere resolution produces two linear replicons with hp telomeres.

Predicted Alignment Error Plot for TelA model

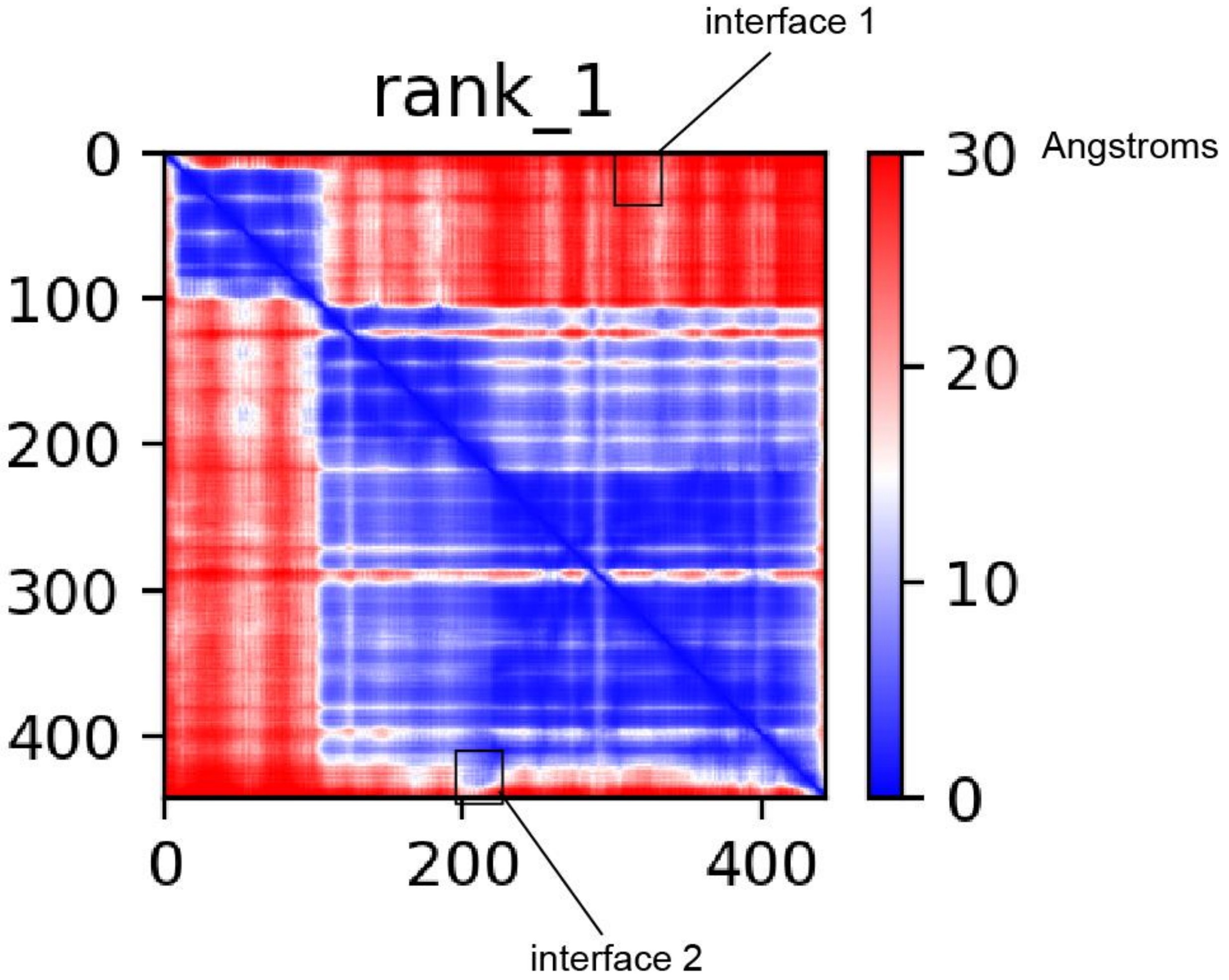

S2 Fig. Plot of the Predicted Aligned Error estimates for the top ranked AlphaFold2 model for TelA.

Heat map of the predicted aligned error (PAE) of the top ranked AlphaFold2 model used in Figure 1 of this study. The units are Anstroms and a low number indicates a high confidence and a high number indicates low confidence. The position of the hypothesized interfaces is highlighted by labeled boxes on the PAE plot.

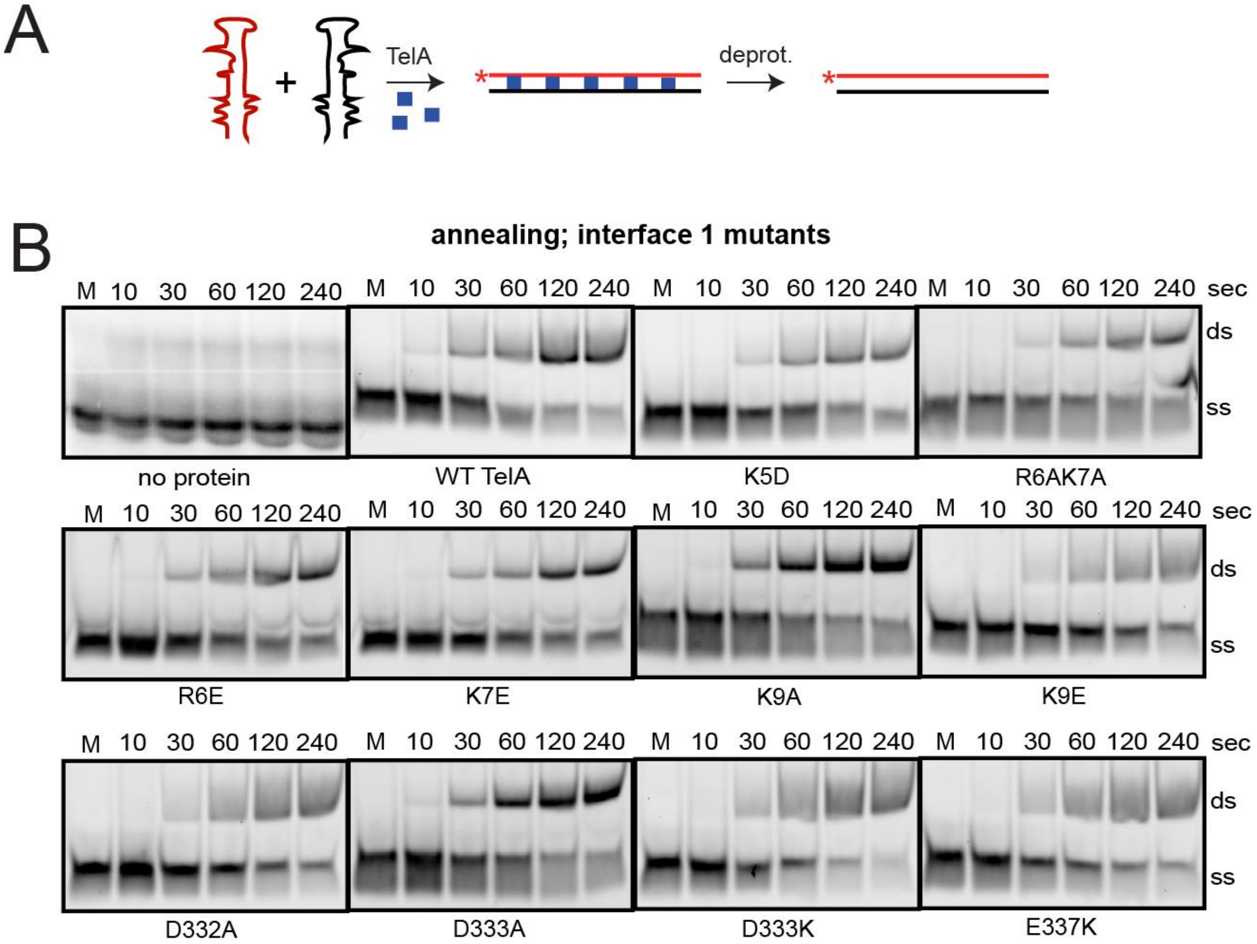

S3 Fig. Single-stranded DNA annealing of interface 1 mutants.

A) Graphical summary of the single-stranded DNA annealing assays using the highly structured HIV_{TAR} element. The 5'-fluorescein endlabeled reporter strand is shown in red and the complementary, unlabeled strand is shown in black. The deproteinated double-stranded product is visualized by polyacrylamide gel electrophoresis.

B) 8% PAGE 1X TAE/0.1% SDS gel panels of annealing timecourses with the indicated interface 1 TelA mutants. Annealing reactions are as reported in the Supplementary Methods.

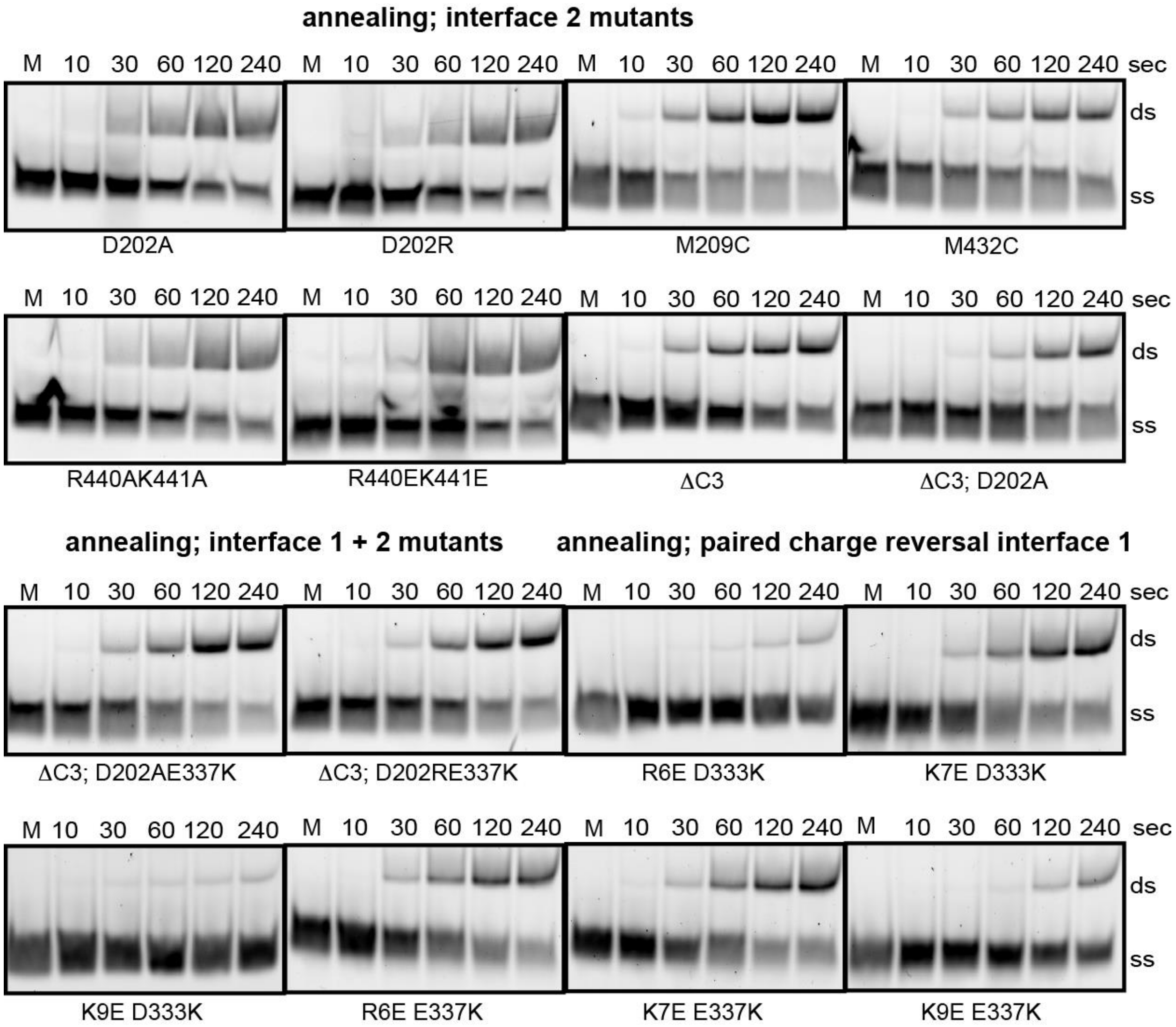

S4 Fig. Single-stranded DNA annealing of interface 2, interface 1 + 2 double mutants and interface 1 paired charge mutants.

8% PAGE 1X TAE/0.1% SDS gel panels of annealing timecourses with the indicated TelA mutants.

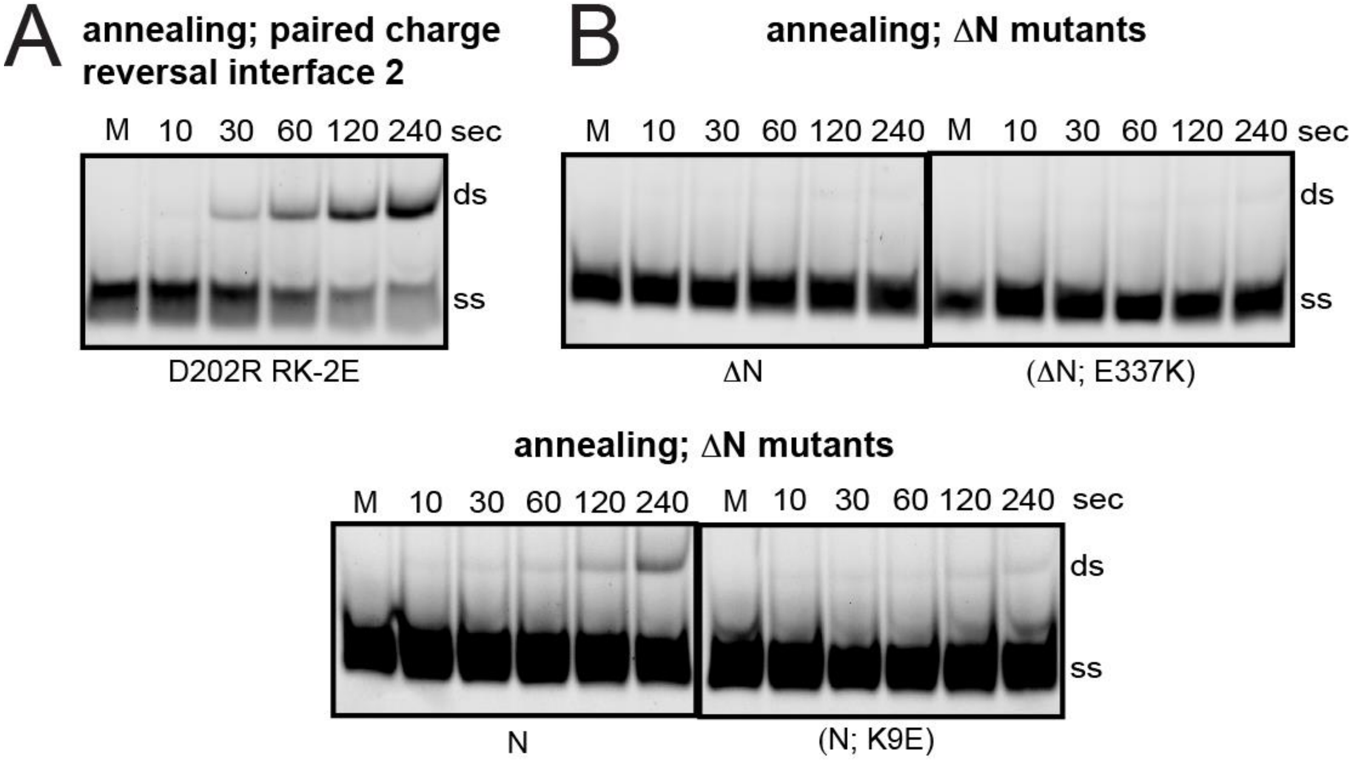

S5 Fig. Single-stranded DNA annealing of interface 2 paired charge reversal mutant and N-terminal domain deletion mutants.

A) 8% PAGE 1X TAE/0.1% SDS gel panel of an annealing timecourse with the D202R R440EK441E mutant.

B) 8% PAGE 1X TAE/0.1% SDS gel panels of annealing timecourses with the indicated N-terminal domain deletion mutants. ΔN denotes TelA (107-442) with a deletion of the N-terminal domain, (ΔN; E337K) denotes the N-terminal domain deletion paired with the activating E337K mutation, N denotes TelA (1-106) and (N; K9E) denotes the TelA (1-106) paired with the activating mutation K9E.

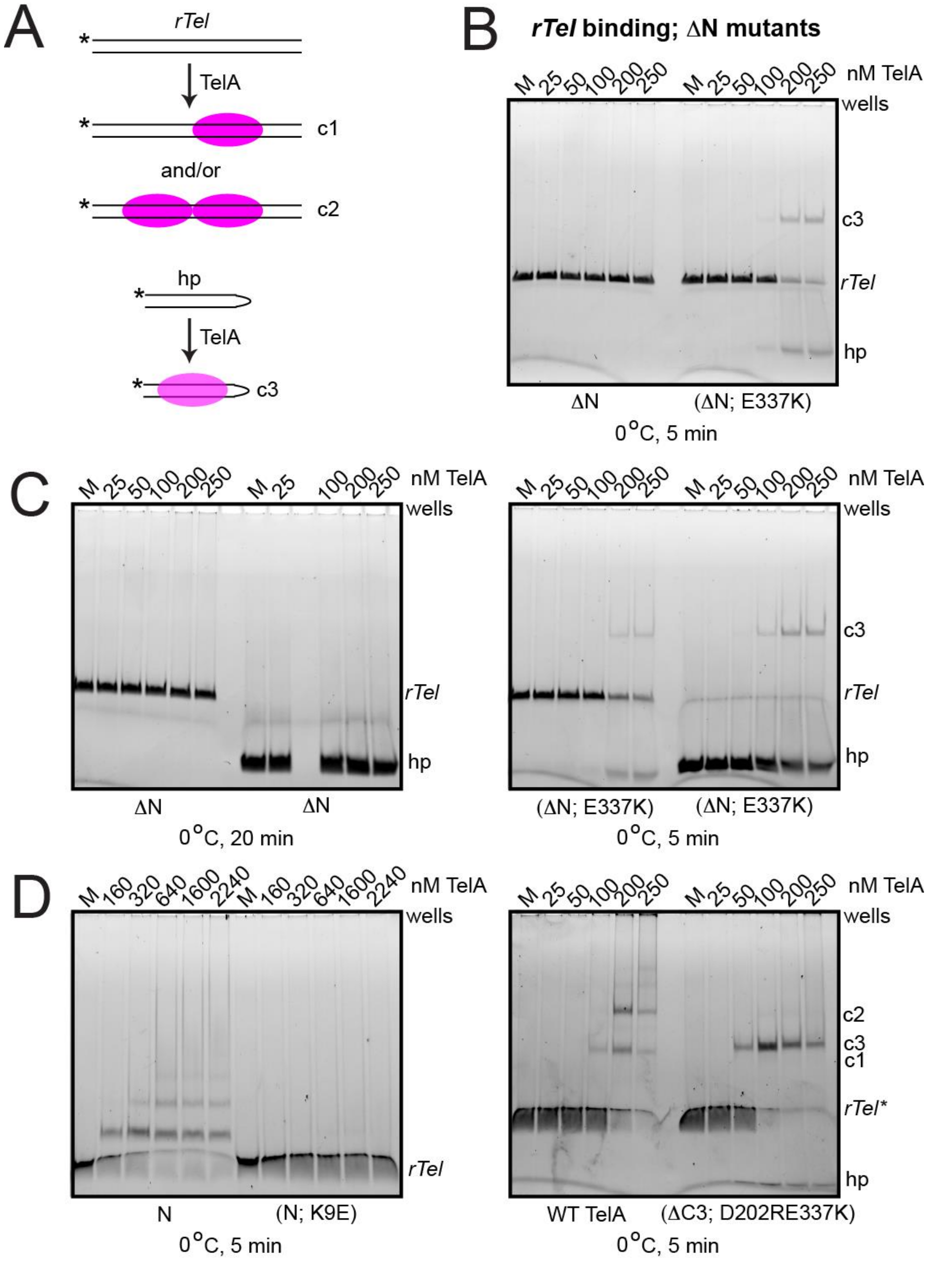

S6 Fig. Electrophoretic mobility shift assays (EMSA) of the N-terminal domain and the N-terminal domain deletion mutants.

A) Graphic of the EMSA assay of TelA with a replicated telomere junction (*rTel*) *vs*. a hp product. * indicate the position of the 5’-fluorescein endlabel; *rTel* denotes the replicated telomere substrate; hp denotes the hairpin telomere product and c1-3 denote the inferred structure of the observed bandshifts.

B) 6% PAGE 0.5X TBE gel panel of the ΔN and (ΔN; E337K) mutants with a 5’-fluorescein endlabeled *rTel* junction.

C) 6% PAGE 0.5X TBE gel panels of the ΔN (left panel) and (ΔN; E337K) mutants with an *rTel*

junction and a 5'-fluorescein endlabeled hairpin telomere (hp). The TelA-DNA bandshifted species is inferred to be derived from the hp telomere products of the reaction.
D) 6% PAGE 0.5X TBE gel panels of the N-terminal domain (N) and N-terminal domain with the K9E mutation (N; K9E) in the left panel. The right gel panel shows wild type (WT) TelA and the ∆C3 truncation combined with the activating D202R and E337K point mutations (∆C3; D202RE337K). In both cases, the *rTel* used incorporated a T to C mutation at the first nucleotide between the scissile phosphates. This *rTel* was designed to slow reaction to try and obtain *rTel* bandshifts instead of just hp product bandshifts. *rTel** denotes use of this mutant *rTel*.

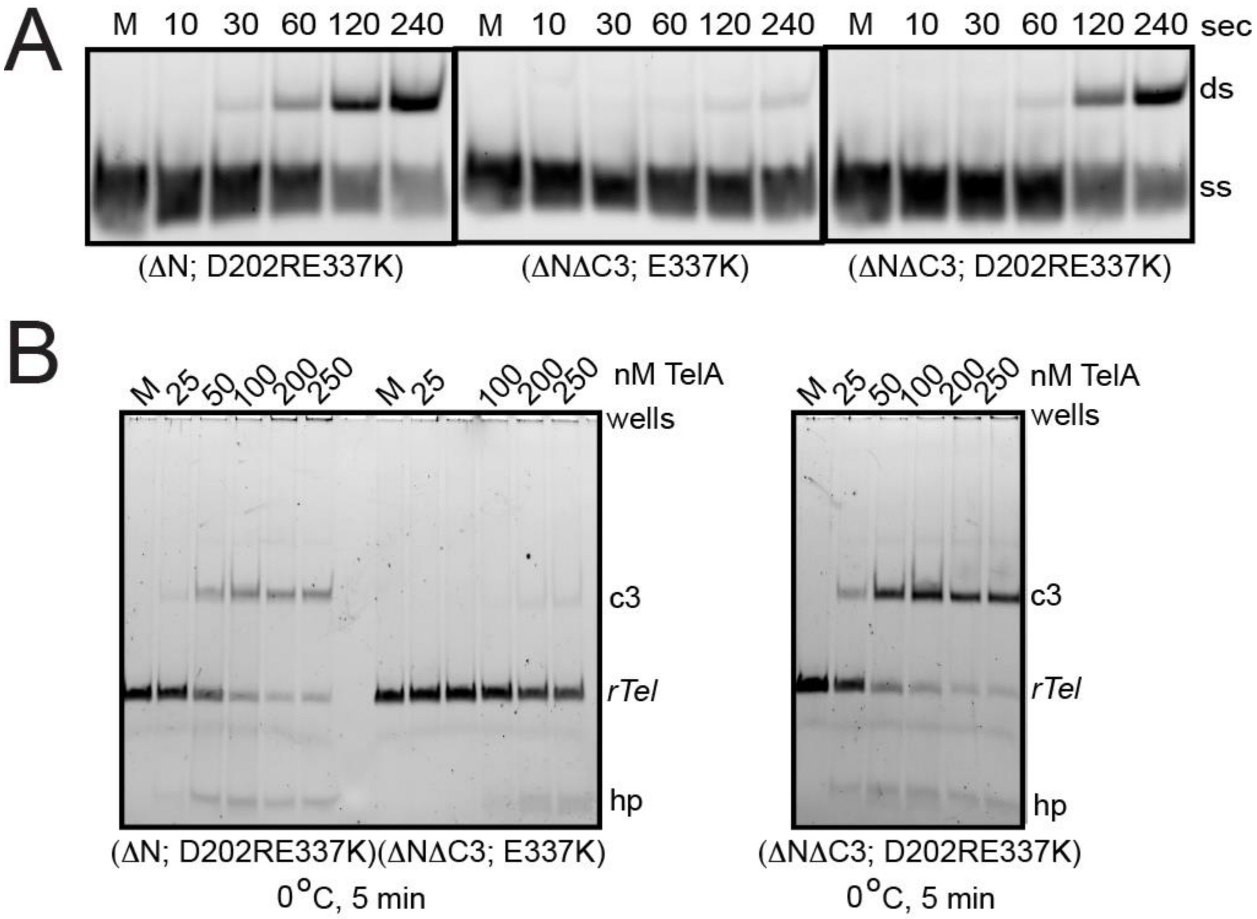

S7 Fig. Electrophoretic mobility shift assays (EMSA) of the TelA mutants that combine activating mutations.
A) 8% PAGE 1X TAE/0.1% SDS gel panel of an annealing timecourse with the indicated mutants.
B) 6% PAGE 0.5X TBE gel panel of the indicated mutants with a 5'-fluorescein endlabeled *rTel* junction.

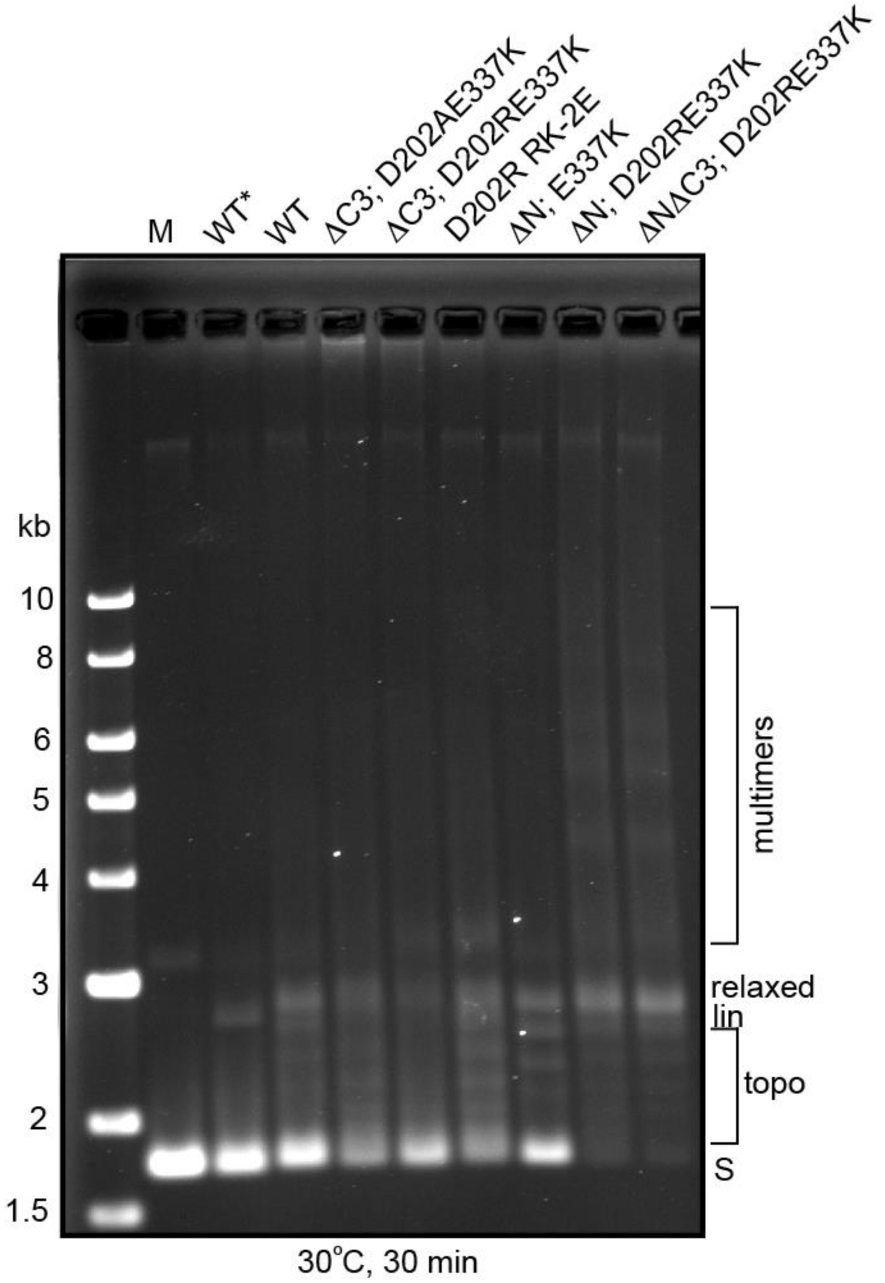

S8 Fig. Hyperactive TelA mutants display topoisomerase activity with a mutant *rTel* plasmid.

0.8% agarose 1X TAE gel panel documenting the reaction of the hyperactive TelA mutants with a plasmid substrate harbouring a mutant *rTel* that inhibits hairpin telomere formation (pEKK495). Incubations were with 76 nM of the indicated proteins and 2 μg/ml plasmid substrate at 30°C for 30 min. S denotes the supercoiled plasmid substrate; lin denotes the migration position of linear DNA; relaxed the position of fully relaxed plasmid.

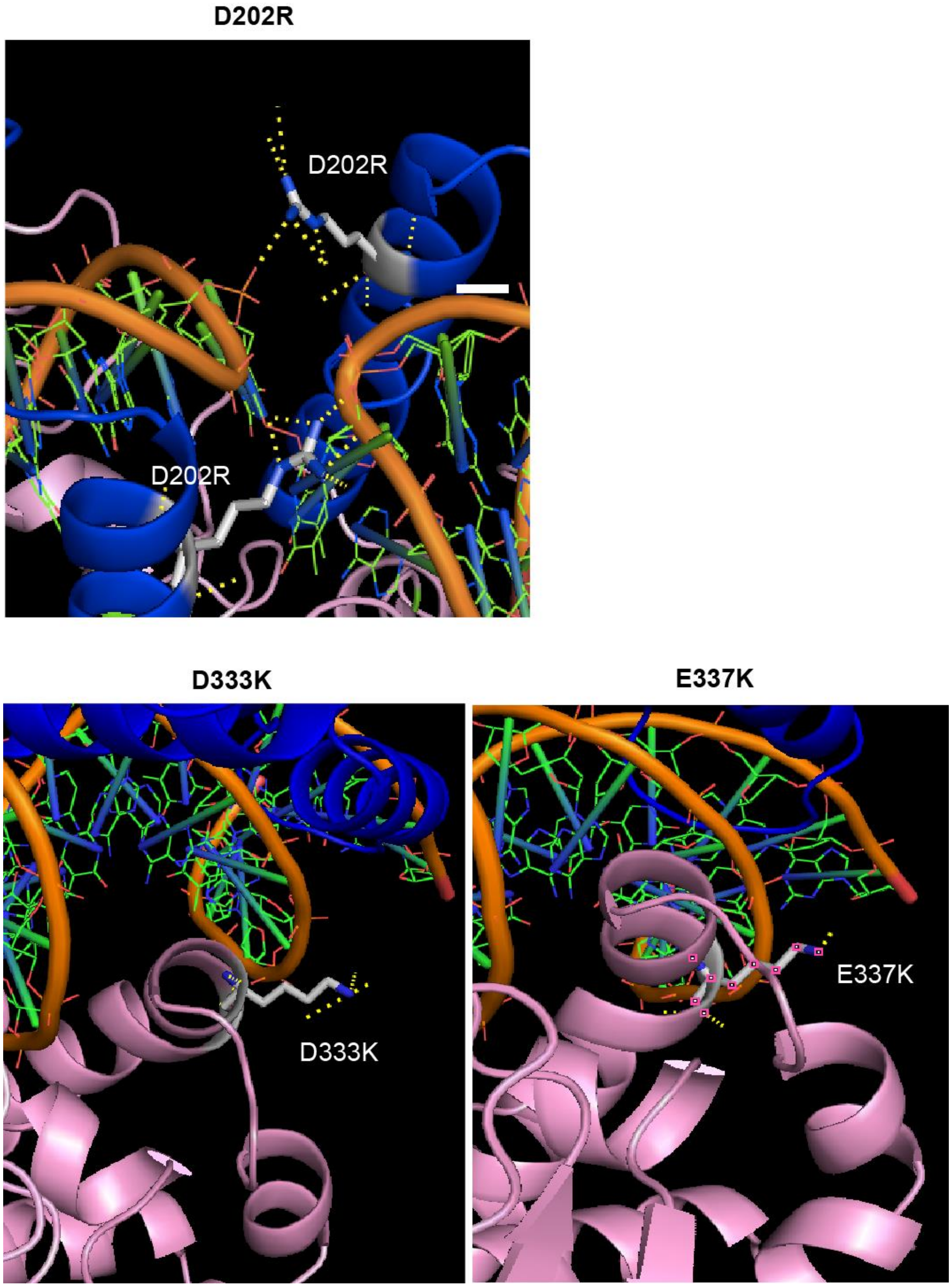

S9 Fig. Analysis of digital mutants D202R, D333K and E337K for creation of polar contacts with substrate DNA.

Using the structure of TelA complexed with product DNA (PDB accession # 4e0g) the D202, D333 and E337 sidechains were replaced in the structures with positively charged sidechains. The ability of these mutations to introduce polar contacts with the substrate DNA was assessed using PyMol.

S1 Table. Oligonucleotides used in this study.

Oligo name	Oligo sequence	Use
OKBA39	5'-ggcgtttttgtcttacgatcggccgccagcatatggc-3'	Make K5D; top strand; WT parent
OKBA40	5'-gccatatgctggcggccgatcgtaagacaaaaacgcc-3'	Make K5D; bot. Strand; WT parent
OKBA33	5'-acactggcgtttttgtcttctccttggccgccagcatatgg-3'	Make R6E; ts; WT parent
OKBA34	5'-ccatatgctggcggccaaggagaagacaaaaacgccagtgt-3'	Make R6E; bs; WT parent
OKBA35	5'-ggcgtttttgtctcacgcttggccgccagc-3'	Make K7E; ts; WT parent
OKBA36	5'-gctggcggccaagcgtgagacaaaaacgcc-3'	Make K7E; bs; WT parent
OGCB989	5'-GCGGCCAAGCGTAAGACAGCGACGCCAGTGTTAGTGTTA-3'	Make K9A; ts; WT parent
OGCB990	5'-TAACACTAACACTGGCGTCGCTGTCTTACGCTTGGCCGC-3'	Make K9A; bs; WT parent
OKBA15	5'-aacactggcgtctctgtcttacgcttggccgccag-3'	Make K9E; ts; WT parent
OKBA16	5'-ctggcggccaagcgtaagacagagacgccagtgtt-3'	Make K9E; bs; WT parent
OKBA47	5'-cggacgagaagtcggcgatcgacatgcca-3'	Make D332A; ts; WT parent
OKBA48	5'-tggcatgtcgatcgccgacttctcgtccg-3'	Make D332A; bs; WT parent
OGCB991	5'-GGCATGTCGATCGACGCCTTCTCGTCCGAGACA -3'	Make D333A; ts; WT parent
OGCB992	5'-TGTCTCGGACGAGAAGGCGTCGATCGACATGCC-3'	Make D333A; bs; WT parent
OKBA17	5'-gtgtctcggacgagaacttgtcgatcgacatgcca-3'	Make D333K; ts; WT parent

OKBA18	5'-tggcatgtcgatcgacaagttctcgtccgagacac-3'	Make D333K; bs; WT parent
OGCB993	5'-GACGACTTCTCGTCCGCGACACGTCTGCTGTTA-3'	Make E337A; ts; WT parent
OGCB994	5'-TAACAGCAGACGTGTCGCGGACGAGAAGTCGTC-3'	Make E337A; bs; WT parent
OKBA19	5'-acagcagacgtgtcttggacgagaagtcgtc-3'	Make E337K; ts; WT parent
OKBA20	5'-gacgacttctcgtccaagacacgtctgctgt-3'	Make E337K; bs; WT parent
OGCB772	5′-GCTGCTATGTACGCGGAAGCTCGTCGT-3′	Make D202A; ts; WT parent
OGCB773	5′-ACGACGAGCTTCCGCGTACATAGCAGC-3′	Make D202A; b; WT parents
OKBA37	5'-aacacgacgagcttcgcggtacatagcagcgtcg-3'	Make D202R; ts; WT parent
OKBA38	5'-cgacgctgctatgtaccgcgaagctcgtcgtgtt-3'	Make D202R; bs; WT parent
OGCB995	5'-GACGAAGCTCGTCGTGTTAAGTGCGAAAAAATCGCGAATAAACAC-3′	Make M209C; ts; WT parent
OGCB996	5'-GTGTTTATTCGCGATTTTTTCGCACTTAACACGACGAGCTTCGTC-3'	Make M209C; bs; WT parent
OGCB997	5'-CGCACATTGCAACAGTGCGCCACCATTGCGCCC-3′	Make M432C; ts
OGCB998	5'-GGGCGCAATGGTGGCGCACTGTTGCAATGTGCG-3'	Make M432C; bs; WT parent
OGCB997	5'-CGCACATTGCAACAGTGCGCCACCATTGCGCCC-3'	Make M209CM432C; ts; M209C parent
OGCB998	5'-GGGCGCAATGGTGGCGCACTGTTGCAATGTGCG-3'	Make M209CM432C; bs; M209C parent

OGCB964	5'-GCGCCCGTAAGTGCTGCGGGGTAAGGATCC-3'	Make R440AK441A; ts; WT parent
OGCB965	5'-GGATCCTTACCCCGCAGCACTTACGGGCGC-3'	Make R440AK441A; bs; WT parent
OKBA13	5'-tgcgccaccattgcgcccgtaagtgaggaggggtaaggatc-3'	Make R440EK441E; ts; WT parent
OKBA14	5'-gatccttacccctcctcacttacgggcgcaatggtggcgca-3'	Make R440EK441E; bs; WT parent
OGCB962	5'-ATTGCGCCCGTAAGTTGAAAGGGGTAAGGATCC-3'	Make TelA (1-439); ts; WT parent
OGCB963	5'-GGATCCTTACCCCTTTCAACTTACGGGCGCAAT-3'	Make TelA (1-439); bs; WT parent
OGCB772	5'-GCTGCTATGTACGCGGAAGCTCGTCGT-3'	Make ∆C3; D202A; ts; ∆C3
OGCB773	5'-ACGACGAGCTTCCGCGTACATAGCAGC-3'	Make ∆C3; D202A; bs; ∆C3
OKBA19	5'-acagcagacgtgtcttggacgagaagtcgtc-3'	Make ∆C3; D202AE337K; ts; ∆C3; D202A parent
OKBA20	5'-gacgacttctcgtccaagacacgtctgctgt-3'	Make ∆C3; D202AE337K; bs; ∆C3; D202A parent
OKBA43	5'-aacacgacgagcttccctgtacatagcagcgtcg-3'	Make ∆C3; D202RE337K; ts
OKBA44	5'-cgacgctgctatgtacagggaagctcgtcgtgtt-3'	Make ∆C3; D202RE337K; bs

OGCB795	5’-CACCATGGCATATGGGAGTGGCGACCTCTATCGTTGAAAAG-3’	Make TelA (107-442); ts; WT parent
OGCB794	5’-ATACCGGATCCTTACCCCTTACGACTTACGGGCGC-3’	Make TelA (107-442); bs; WT parent
OKBA19	5'-acagcagacgtgtcttggacgagaagtcgtc-3'	Make ∆N; E337K; ts; ∆N parent
OKBA20	5'-gacgacttctcgtccaagacacgtctgctgt-3'	Make ∆N; E337K; bs; ∆N parent
OKBA15	5'-aacactggcgtctctgtcttacgcttggccgccag-3'	Make N; K9E; ts; N parent
OKBA16	5'-ctggcggccaagcgtaagacagagacgccagtgtt-3'	Make N; K9E; bs; N parent
OKBA37	5'-aacacgacgagcttcgcggtacatagcagcgtcg-3'	Make ∆N; D202RE337K; ts; ∆N; E337K parent
OKBA38	5'-cgacgctgctatgtaccgcgaagctcgtcgtgtt-3'	Make ∆N; D202RE337K; bs; ∆N; E337K parent
OGCB962	5'-ATTGCGCCCGTAAGTTGAAAGGGGTAAGGATCC-3'	Make ∆N; E337K; ∆C3; ts; ∆N; E337K parent
OGCB963	5'-GGATCCTTACCCCTTTCAACTTACGGGCGCAAT-3'	Make ∆N; E337K; ∆C3; bs; ∆N; E337K parent
OKBA27F	5’F-atcCCTCTAACCATTGCGCGATCGATCATAATAACAATATCA-3’	5’-fluorescein labeled ts half-site
OGCB871	5’-TGATATTGTTATTATGATCGATCGCGCAATGGTTAGAGG-3’	bs half-site

OKBA28F	5′-atcCCTCTAACCATTGCGCGATCGATCATAATAAC AATATCATGATATTGTTATTATGATCGATCGCGCAATGGTTAGAGG-3′	5’-fluorescein labeled hairpin telomere
OKBA29F	5′-atcGGAGATTGGTAACGCGCTAGCATGTATTATTGTTATAGT-3′	5’-fluorescein labeled ts mock half-site
OGCB914	5′-CCTCTAACCATTGCGCGATCGTACATAATAACAATATCA-3′	bs mock half-site
OKBA30F	5′-atcGGAGATTGGTAACGCGCTAGCATGTATTATTG TTATAGTACTATAACAATAATACTAGCATGCGCGTTACCAATCTCC-3′	5’-fluorescein labeled mock hairpin telomere
OGCB951 F	5′-aaCTCTAACCATTGCGCGATCGATCATAATAACAA TATCATGATATTGTTATTGTAATCGATCGCGGATCCCGGGCGTAGCCACGTAGGT -3′	5’-fluorescein labeled ts *rTel*
OGCB952	5′-gaACCTACGTGGCTACGCCCGGGATCCGCGATCGA TTACAATAACAATATCATGATATTGTTATTATGATCGATCGCGCAATGGTTAGAG -3′	bs *rTel*
OKBA45F	5′-aaGAGATTGGTAACGCGCTAGCTAGTATTATTGTT ATAGTACTATAACAATAACATTAGCTAGCGCCTAGGGCCCGCATCGGTGCATCCA -3′	5’-fluorescein labeled ts mock *rTel*
OKBA46	5′-gaTGGATGCACCGATGCGGGCCCTAGGCGCTAGCT AATGTTATTGTTATAGTACTATAACAATAATACTAGCTAGCGCGTTACCAATCTC -3′	bs mock *rTel*
OGCB951	5′-aaCTCTAACCATTGCGCGATCGATCATAATAACAA TATCATGATATTGTTATTGTAATCGATCGCGGATCCCGGGCGTAGCCACGTAGGT -3′	ts *rTel*
OKBA45	5′-aaGAGATTGGTAACGCGCTAGCTAGTATTATTGTT ATAGTACTATAACAATAACATTAGCTAGCGCCTAGGGCCCGCATCGGTGCATCCA -3′	ts mock *rTel*

F refers to IDT’s 5’-FAM modification for a fluorescein label. Sequence shown in lowercase for oligonucleotides used to anneal substrate DNA’s represent overhangs in the resulting substrates.

S2 Table. Induction/expression conditions for TelA mutants.

Strain number	Mutant	Induction/expression conditions
EKK543	K5D	0.25 mM IPTG induction at 24°C O/N
EKK542	R6E	0.5 mM IPTG induction at 24°C O/N
EKK539	K7E	0.5 mM IPTG induction at 24°C O/N
EKK511	K9A	0.5 mM IPTG induction at 24°C O/N
EKK526	K9E	0.5 mM IPTG induction at 24°C O/N
EKK560	D332A	0.25 mM IPTG induction at 24°C O/N
EKK512	D333A	0.5 mM IPTG induction at 24°C O/N
EKK529	D333K	0.25 mM IPTG induction at 24°C O/N
EKK514	E337A	0.125 mM IPTG inductions at 24°C O/N
EKK527	E337K	0.25 mM IPTG induction at 24°C O/N
EKK401	D202A	0.25 mM IPTG induction at 24°C O/N
EKK540	D202R	0.25 mM IPTG induction at 24°C O/N
EKK513	M209C	0.5 mM IPTG induction at 24°C O/N
EKK506	M432C	0.25 mM IPTG induction at 24°C O/N
EKK528	M209CM432C	0.125 mM IPTG inductions at 24°C O/N
EKK471	R440AK441A (RK-2A)	0.5 mM IPTG 6h induction at 24°C
EKK515	R440EK441E (RK-2E)	0.25 mM IPTG induction at 24°C O/N
EKK470	TelA (1-439) ΔC3	0.25 mM IPTG induction at 24°C O/N
EKK566	R6ED333K	0.25 mM IPTG induction at 24°C O/N
EKK554	K7ED333K	0.125 mM IPTG inductions at 24°C O/N
EKK570	K9ED333K	0.25 mM IPTG induction at 24°C O/N
EKK557	R6EE337K	0.25 mM IPTG induction at 24°C O/N
EKK549	K7EE337K	0.25 mM IPTG induction at 24°C O/N
EKK555	K9EE337K	0.25 mM IPTG induction at 24°C O/N
EKK482	ΔC3; D202A	0.5 mM IPTG induction at 24°C O/N

EKK538	ΔC3; D202AE337K	0.25 mM IPTG induction at 24°C O/N
EKK556	ΔC3; D202RE337K	0.25 mM IPTG induction at 24°C O/N
EKK408	TelA (107-442) ΔN	0.5 mM IPTG induction at 24°C O/N
EKK567	ΔN; E337K	0.125 mM IPTG inductions at 24°C O/N
EKK571	N; K9E	0.25 mM IPTG induction at 24°C O/N
EKK577	ΔN; D202RE337K	0.25 mM IPTG induction at 24°C 6h
EKK578	ΔNΔC3; E337K	0.25 mM IPTG induction at 24°C 6h
EKK581	ΔNΔC3; D202RE337K	0.25 mM IPTG induction at 24°C 6h

Supplemental Methods

Electrophoretic mobility shift assays (EMSA)

TelA was bandshifted with its substrate *rTel* or hp product in a buffer containing 25 mM HEPES (pH 7.6), 1 mM DTT, 2 mM $CaCl_2$, 100 μg/mL BSA, 50 mM potassium glutamate, 0.8 μg/mL competitor DNA (supercoiled pUC19) and 76 ng/mL heparin sulphate. The indicated concentrations of TelA were incubated at 0°C for the times indicated beneath the gel panels with 2 nM of 5' fluorescein-endlabeled *rTel* assembled from oligonucleotides OGCB951F/952 (see S1 Table). After the native load dye was added to a 1X concentration, samples were applieded to 6% PAGE 0.5X Tris-Borate EDTA (TBE) gels followed by electrophoresis at 15V/cm in a 4°C until the dye front was 1 cm from the gel bottom. The gels were visualized for documentation on a BioRad GelDoc system using the UV transilluminator and the fluorescein program. 1X loading dye contains 20 mM EDTA, 3.2% glycerol, and 0.024% bromophenol blue.

ssDNA annealing assays

The annealing assays were performed in a buffer containing 25 mM HEPES (pH 7.6), 1 mM DTT, 2 mM $CaCl_2$, 100 μg/mL BSA, 50 mM potassium glutamate and 15 nM of the 5' fluorescein-endlabeled reporter oligonucleotide (OKBA51F; S1 Table). To prevent spontaneous annealing the reactions were assembled on ice prior to addition of 15 nM the complementary oligonucleotide (OGCB899) and 154 nM TelA. Following the addition of TelA the reactions were incubated at 30°C. 18 μL aliquots were taken from the annealing reactions at the indicated timepoints. To stop further annealing, these aliquots were resuspended in an SDS-containing load dye to a 1X final concentration that contained 0.3 μM of the unlabeled reporter oligonucleotide (OKBA51). 1X load dye contains 0.15% SDS, 20 mM EDTA, 3.2% glycerol, and 0.024% bromophenol blue. The oligonucleotides used for the annealing assay are derived

from the HIV_{TAR} element [1]. Where the N-terminal domain is used in annealing assays 800 nM of N was added to the reactions.

Supplemental References

1. Lapadat-Tapolsky M PC, Borie C and Darlix J-L. Analysis of the nucleic acid annealing activities of nucleocapsid protein from HIV-1. Nucleic Acids Research. 1995;23(1):2434-41.
