# Supplementary material for: Design and characterization of hyperactive mutants of the Agrobacterium tumefaciens telomere resolvase, TelA
Source: PLoS One. 2024 Jul 25;19(7):e0307590. doi: 10.1371/journal.pone.0307590 (PMC11271964; doi:10.1371/journal.pone.0307590)
Supplement: Attachment — Submitted filename: Response to Reviewers.docx [file pone.0307590.s003.docx]

**Response to Reviewers:**

Dear Dr. Lustig,

We thank you and the two reviewers for their expert evaluation of our manuscript ‘Design and Characterization of Hyperactive Mutants of the *Agrobacterium tumefaciens Telomere Resolvase, TelA.’ (*ONE-D-24-17544). We feel that addressing the reviewer comments/concerns has markedly strengthened the revised manuscript. In brief, we have added a Supplementary Figure detailing the predicted aligned error of the model used in the paper (S2 Fig). A second Supplementary Figure (S9 Fig) details the results of digital mutagenesis to assess introduction of polar contacts with D202R, D333K and E337K mutations with DNA potentially caused by these charge reversal mutations. We have also added an additional data figure in the main text of the paper (Figure 8) wherein we test the two most useful hyperactive mutants against wild type TelA at the standard reaction temperature of 30^o^C (instead of 0^o^C) using a low protein concentration to slow reactions for ease of measuring initial rates (3 nM as opposed to 74 nM). The mutants chosen for analysis were hyperactivated (>50-fold) but retained enough autoinhibition to suppress competing reactions. These changes have necessitated the renumbering of supplementary figure references and has lengthened, somewhat, the manuscript to incorporate discussion of this new figure and also discussion of the predicted aligned error results. What follows below is a point by point response to the reviewer/editor comments.

Editor comments:

Based on the critiques of reviewers and of my independent reading, the following comments need to be addressed:

1. I concur with Reviewer 1 that a score be presented the best fit AlphaFold model. A poor model might explain the lack of data support. This does not take of course take away from the interesting effects of the mutants just the logical framework of the data presentation.

   We have prepared a new supplementary Figure with the Predicted Aligned Error (S2 Fig). This has necessitated the renumbering of the SFig referents throughout text. We have discussed the error estimates in the results and discussion sections. The take home is that the predicted aligned error estimates reveal that the model is low confidence for interface 1 and much higher confidence for interface 2. Nonetheless, testing both predicted interfaces proved a useful exercise for uncovering activating mutations.
3. Reviewer 1 also suggests testing in a subset of mutants, in which activity is varied by altering the concentration of the enzyme.  Either conduct such an experiment for the most hyperactive mutant or provide a rebuttal as to why you feel that this is uncessary.

   This was an excellent suggestion since the specific ranking of activities could be a read out of general activation but also, potentially, a read out of the effect of a mutation specifically on the cold-sensitivity of telomere resolution. We repeated telomere resolution assays for WT, (ΔC3; D202AE337K) and (ΔN; E337K) at 30^o^C at low protein concentration (3 nM). These two mutants were selected since they do not suffer from activation of inappropriate activities due to their hyperactivation. This analysis has become Fig. 8. The results indicate that activation is also seen at 30^o^C for these two mutants but the relative ranking of their activities has changed indicating that there **were** differential effects on the cold-sensitivity of the telomere resolution.

   Based on my reading of the text, I suggest the following additional changes to improve the clarity of the paper.

   Page 12, line 9: It is unclear to me whether these events behave in a strictly additive way. To Please provide the expectations of additivity vs the results obtained within the text.

   What is meant by additive is now spelled out and a counter example of non-additive behaviour of a double mutant is provided if the mutations affect a common autoinhibitory interface. It essentially boils down to something akin to an *in vitro* epistasis analysis.
4. Page 13, Line 4. You say that D202 and deltaC-3 are additive. Please explain this outcome. Was this outcome expected?

   We have clarified this section by indicating what our expectations when combining the D202 mutants with the delta-C3 mutation. The increased activation observed was unexpected and foreshadowed the result of the more rigorous paired charge reversal results documented later in the paper that these residues are unlikely to interact to help form the modeled interface.
5. Phenotypic separation: Since you discuss annealing activity and other enzyme characteristics at multiple points within the text, it would be very helpful to provide a summary table of all of the characteristics of each mutant/activity.

   Such a mass of different assay results applied to numerous mutants has now been made more accessible, at a glance, by creation of Table 1 for the main body of the paper.
6. Redefine hp and rTel when first used in the Results section for clarity.

   Completed as requested to improve clarity of presentation of the results.
7. Figure 4, how many replicates of each mutant ws used to derive the mean and standard deviation.

   We have changed all figure legends to indicate that the graphed results show the mean and standard deviation of 3 independent replicates excepting the noted mutants that were assayed with 6 replicates. This affords greater clarity.

Reviewer #1 comments:

*1) The AlphaFold prediction of DNA-unbound TelA served as the template for designing the mutants analyzed in this work. Although the individual domain structures are likely to be accurately predicted, it’s not clear if the domain-domain contacts were predicted with high confidence. The authors should show and discuss error estimates in the prediction, especially the Predicted Aligned Error.*

We have prepared a new supplementary Figure with the Predicted Aligned Error (S2 Fig). This has necessitated the renumbering of the SFig referents throughout text. We have discussed the error estimates in the results and discussion sections. The take home is that the predicted aligned error estimates reveal that the model is low confidence for interface 1 and much higher confidence for interface 2. Nonetheless, testing both predicted interfaces proved a useful exercise for uncovering activating mutations.
 *Also, the original paper reporting AlphaFold (PMID: 34265844) should be cited.*

The relevant Jumper *et al.* reference was in the results text but has also been added to the legend for Figure 1.

2) *Most hairpin resolution assays were done at 0 deg, which is unusual. Data shown in Fig. 5B (WT vs. delta N) suggest that an enzyme completely inactive at 0 deg can be more active than WT at 30 deg. I don’t recommend repeating everything at 30 deg., but some of the most interesting mutants’ data (e.g., activities of the most active mutant) could be confirmed at 30 deg. It should be possible to slow the reaction by lowering the enzyme concentration, etc.*

This was an excellent suggestion since the specific ranking of activities could be a read out of general activation but also, potentially, a read out of the effect of a mutation on the cold-sensitivity of telomere resolution. We repeated telomere resolution assays for WT, (ΔC3; D202AE337K) and (ΔN; E337K) at 30^o^C at low protein concentration (3 nM). These two mutants were selected since they do not suffer from activation of inappropriate activities due to their hyperactivation. This analysis has become Fig. 8. The results indicate that activation is also seen at 30^o^C for these two mutants but the relative ranking of their activities has changed indicating that there **were** differential effects on the cold-sensitivity of the telomere resolution.

3) *The authors should discuss the potential mechanisms of hyperactive mutants based on the DNA-bound TelA structure. For instance, are the D202R, D333K, and E337K substitutions predicted to generate additional DNA backbone contacts?*

We have performed and documented this ‘digital mutagenesis’ using an available TelA co-crystal structural model. This is now S9 Fig. The results show that D202R makes contacts with the DNA absent in the wild type but that D333K and E337K do not unless a modest conformational change occurs due to these mutations.


Reviewer #2 comments:

1) *Fig S7 – I can see the topoisomers, however the “topo” labelling mentioned in the legend is not on the figure.*

This supplementary figure is now S8 Fig and has the ladder of topoisomers bracketed and labeled as topo as noted in the figure legend. We thank the reviewer for catching this oversight.


We thank the reviewers for helping to strengthen the revised manuscript.

Best regards,

Kerri Kobryn, PhD
Associate Professor
Dept of Biochemistry, Microbiology & Immunology
College of Medicine
University of Saskatchewan
kerri.kobryn@usask.ca
